# New genera of philopotine spider flies (Diptera, Acroceridae) with a key to living and fossil genera

**DOI:** 10.3897/zookeys.127.1824

**Published:** 2011-09-08

**Authors:** Jéssica P. Gillung, Shaun L. Winterton

**Affiliations:** 1 Universidade de São Paulo, Departamento de Zoologia, Instituto de Biociências, São Paulo, SP, Brazil; 2 California State Collection of Arthropods, California Department of Food & Agriculture, Sacramento, California, USA

**Keywords:** Spider fly, Acroceridae, cybertaxonomy

## Abstract

Information on the three previously described species of *Halocoryza* Alluaud is updated and a new species for the genus from Isla Carmen, Sea of Cortés, Baja California Sur, México is described. *Halocoryza whiteheadiana* **sp. n**. was found at UV light on a beach of that island. This species does not fit the profile of the other three species, i.e., living on coralline beach sands, or in the Mangrove intertidal zone. Two alternative possibilities as to why this is so are suggested and a study plan for testing these possibilities is proposed.

## Introduction

Spider flies (Diptera: Acroceridae) are a geographically cosmopolitan group although most species are relatively rarely collected. Most species feed at flowers and are likely important specialized pollinators as suggested by their frequently elongate proboscis (often equal to body length) and nectar feeding habits, although some species have reduced or even vestigial mouthparts (Schlinger 1981, 1987). Adults have a distinctive morphology and a wide diversity of form and colour, but usually with a small head, greatly enlarged lower calypter and swollen abdomen. Larvae are parasitoids of spiders, with a hypermetamorphic life cycle consisting of four instars (Schlinger 1981, 1987, 2009).

**Figure 1.**
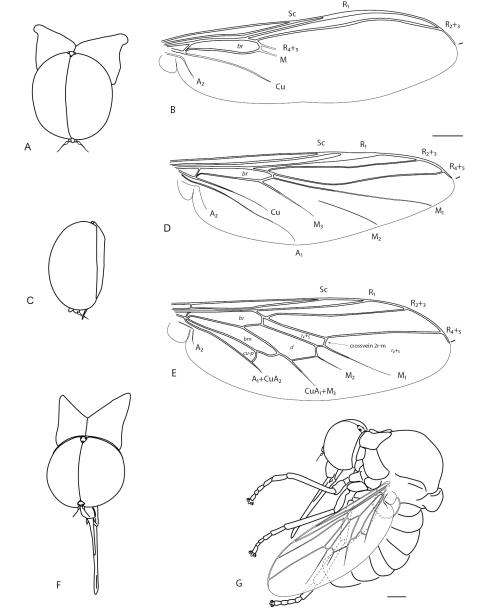
*Quasi fisheri* gen. *et* sp. n. **A** head and postpronotal lobes, anterior **B** wing **C** head, lateral. *Schlingeriella irwini* gen. *et* sp. n. **D** wing. *Eulonchiella eocenica* Meunier **E** head and postpronotal lobes, anterior **F** habitus *in situ*, lateral. Scale line = 0.2 mm.

**Figure 2.**
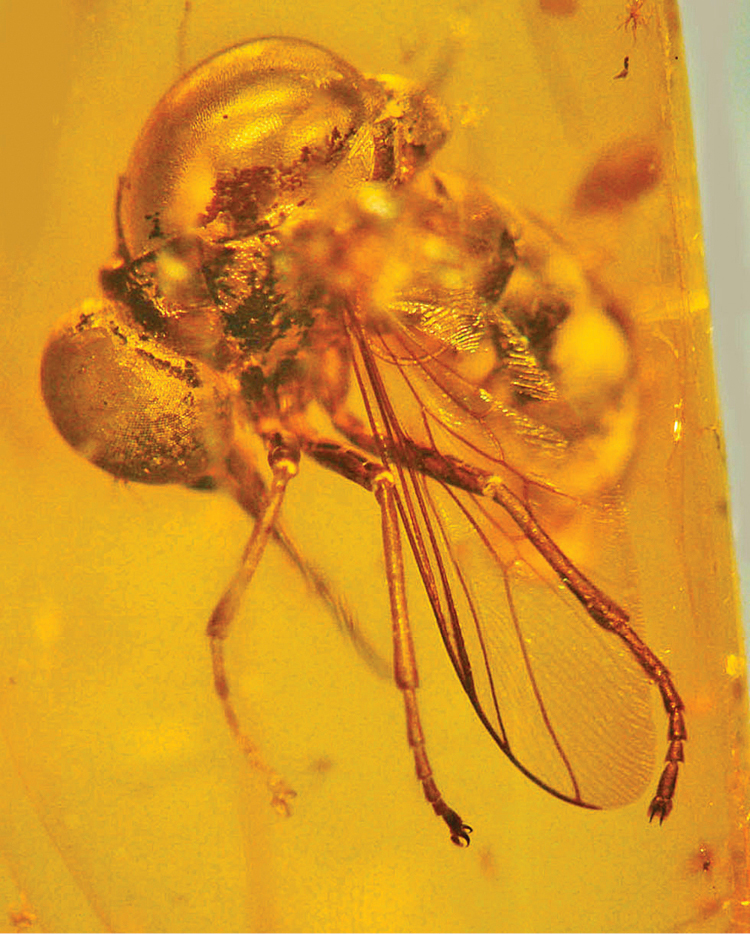
*Eulonchiella eocenica* Meunier (Baltic Amber). Body length = ca. 4.5 mm.

**Figure 3.**
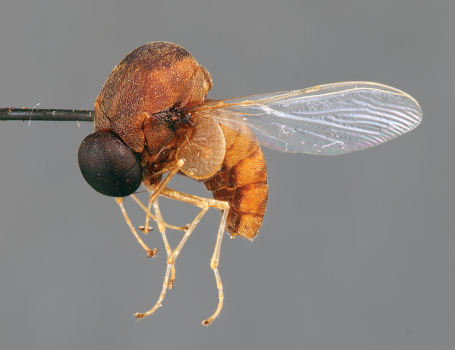
*Quasi fisheri* gen. *et* sp. n., male, anterolateral view [Morphbank: 693076]. Body length = 6.0 mm.

**Figure 4.**
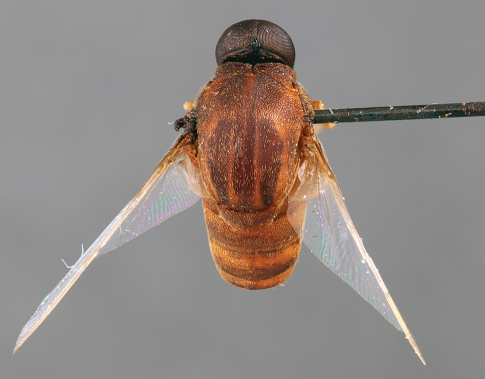
*Quasi fisheri* gen. *et* sp. n., male, dorsal view [Morphbank: 693077]. Body length = 6.0 mm.

**Figure 5.**
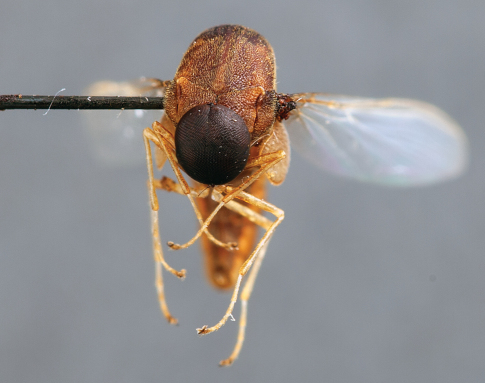
*Quasi fisheri* gen. *et* sp. n., male, anterior view [Morphbank: 693078]. Body length = 6.0 mm.

**Figure 6.**
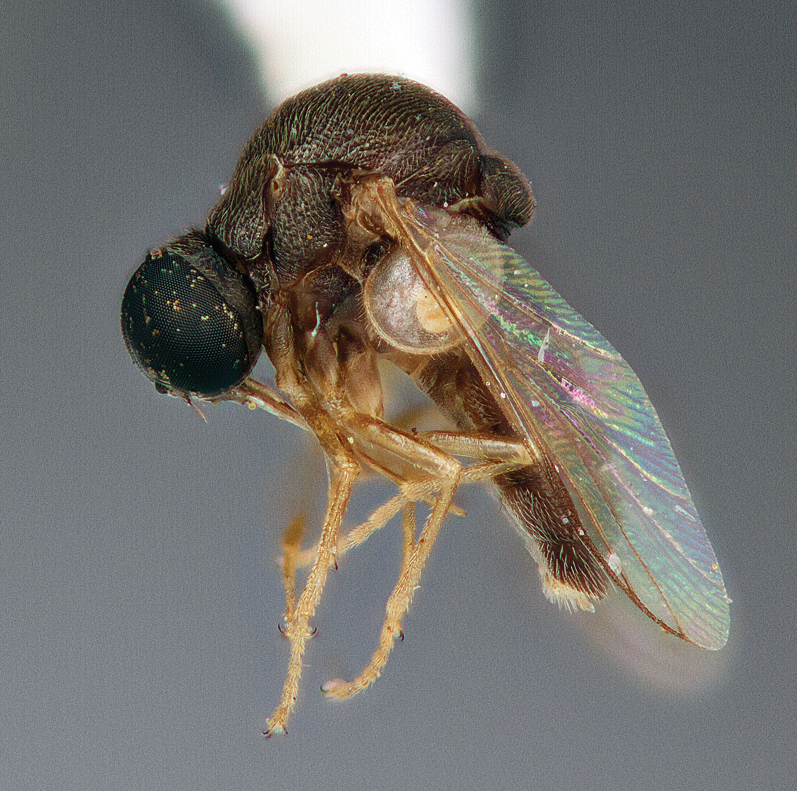
*Schlingeriella irwini* gen. *et* sp. n., male, lateral view [Morphbank: 693079]. Body length = 2.4 mm.

**Figure 7.**
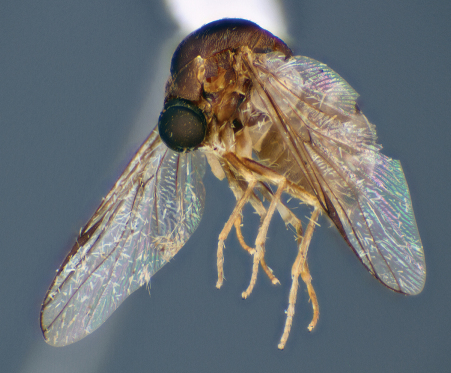
*Schlingeriella irwini* gen. *et* sp. n., female, lateral view [Morphbank: 693080]. Body length = 4.4 mm.

Acroceridae comprise over 520 described species in about 53 genera (Pape and Thompson 2011). The species are traditionally separated in three extant subfamilies – Acrocerinae, Panopinae and Philopotinae, based on adult morphology and host specificity. Panopinae have been postulated as the most primitive and Acrocerinae the most derived, with Philopotinae supposedly occupying an intermediate position (Schlinger 1987). Phylogenetic analyses by Winterton et al. (2007) based on molecular data, however, do not corroborate this subfamilial arrangement, with Acrocerinae recovered as polyphyletic and Panopinae as a derived clade. The monophyly of Philopotinae has never been questioned based on a series of morphological synapomorphies (Schlinger 1981), a position also strongly supported by analyses of molecular data (Winterton et al. 2007). Adults of Philopotinae are characterized by enlarged postpronotal lobes that are usually contiguous dorsomedially to form a collar around the head, as well as varying degrees of arched body shape (Figs 1–7).

There are approximately 52 species and 14 genera in Philopotinae, both living and fossil, found in all major biogeographical regions. Two morphological groups are easily recognizable in the subfamily based on reduction of wing venation (i.e. number of wing cells and primary veins approximating wing margin). The first group comprises six genera with relatively complete wing venation and includes: *Dimacrocolus* Schlinger, 1961 (Madagascar), *Eulonchiella* Meunier, 1912 (Baltic amber), *Helle* Osten Sacken, 1896 (New Zealand), *Megalybus* Philippi, 1865(Chile), *Parahelle* Schlinger, 1961 (Madagascar) and *Thyllis* Erichson, 1840 (South Africa and Madagascar). The second group comprises eight genera characterized by reduced wing venation such that cells d, bm and even m3 are absent through reduction and loss of crossveins. The wings typically have only major veins radiating from cell br (Figs 1B, D). Genera in this group include *Africaterphis* Schlinger, 1968 (Africa), *Archaeterphis* Hauser & Winterton, 2007 (Baltic amber), *Oligoneura* Bigot, 1878 (Palaearctic), *Philopota* Wiedemann, 1830 (South and Central America), *Prophilopota* Hennig, 1966 (Baltic amber), *Quasi* gen n. (Mexico), *Schlingeriella* gen n. (New Caledonia) and *Terphis* Erichson, 1840 (South America).

*Eulonchiella eocenica* Meunier, 1912 was briefly described and poorly illustrated by Meunier (1910) and (1912) from Baltic amber (only in the latter publication was the name *Eulochiella eocenica* applied for the first time). The holotype was deposited in the Albertus University Collection in Königsberg - Prussian territory at the time and now belonging to Russia - but was lost during the Second World War (Hennig 1966). Fortunately, Frank Hull made relatively more accurate drawings than Meunier and notes about the fossil during a visit to this collection prior to the war. Based on these unpublished data, Hennig (1966) redescribed and figured the species. Recently a specimen in the George Poinar collection, matching the original descriptions by (Meunier (1910, 1912) and subsequent redescription and figure in Hennig (1966), has been identified as *Eulonchiella eocenica*. This individual is also from Baltic amber deposits and is preserved in excellent condition. Herein we diagnose *Eulonchiella eocenica* and designate a neotype based on this newly discovered specimen. We also describe the new genera *Schlingeriella irwini* gen. *et* sp. n. from New Caledonia and *Quasi fisheri* gen. *et* sp. n. from Mexico, and provide a dichotomous key to all living and fossil genera of Philopotinae.

## Materials and methods

Terminology follows McAlpine (1981) and Schlinger (1981). In most acrocerids, two crossveins span the area between the radial and medial sectors enclosing the cell r4+5. The proximal crossvein is r-m, while the distal crossvein bisecting cell r4+5 (between wing veins M1 and R4+5, or rarely R5) is referred to here as 2r-m following Hardy (1946). Collections where specimens are deposited are as follows:Muséum National d’Histoire Naturelle, Paris, France (MNHN), California Academy of Science, San Francisco, USA (CAS), California State Collection of Arthropods, Sacramento, USA (CSCA) and Queensland Museum, Brisbane, Australia (QM). Descriptions were constructed using Lucid Builder 3.5, using a matrix database of character states, which were then exported using the natural language function into XML and a text document. Specimen images were taken at different focal points using a digital camera and subsequently combined into a serial montage image using Helicon Focus software. High-resolution digital images were deposited into Morphbank with embedded URL links within the document between descriptions and Morphbank images. All new nomenclatural acts and literature are registered in Zoobank (Pyle and Michel 2008).

## Taxonomy

### Eulonchiella

Meunier

http://species-id.net/wiki/Eulonchiella

Figs 1E–G 2

Eulonchiella eocenica Meunier 1912: 177 – Meunier 1910: 177, Brunetti 1926: 583, Hennig 1966: 7, Evenhuis 1994: 311. Type species: *Eulonchiella eocenica* Meunier, 1912: 177.

#### Type material.

**Neotype** male, Baltic amber (#DB 10-12) (CAS).

#### Diagnosis.

Body shape arched; colouration non-metallic brown-black; head spherical, size slightly smaller than thorax width; eye bare; male frons narrowed; eyes contiguous above and below antennal base; posterior margin of eye rounded; proboscis length greater than head length; position of antenna in middle of frons; flagellum shape stylate; palpus present; thorax with postpronotal lobes enlarged, medially contiguous to form collar; legs not greatly elongated, tibial spines absent; pulvilli present; subscutellum slightly enlarged; wing hyaline, markings absent; costa ending in radial field; costal margin straight in both sexes; humeral crossvein present; alula well developed; anal lobe not enlarged; R2+3 present; R4+5 present as single vein; radial veins meeting wing margin before wing apex; cell r4+5 bisected by crossvein 2r-m, narrow elongate; discal cell present, closed apically; medial veins M1, M2 and M3 present; medial veins tapered and faint towards margin; cell m3 absent; CuA1 joining M3 and petiolate, not reaching wing margin; CuA2 fused to A1, not reaching wing margin, petiolate; abdomen smooth, shape rounded, cylindrical, similar width to thorax.

#### Comments.

The above diagnosis is based on a neotype male of *Eulonchiella eocenica* Meunier deposited in the Poinar collection (#DB 10-12) (to be ultimately housed in CAS). Hennig (1966) discussed this monotypic genus based on drawings by Meunier (1910) and a drawing provided by Frank Hull (published in Hennig 1966) before the type was destroyed. The enlarged abdomen in the drawing by Hull indicates that the original type was a female. The specimen examined herein is a male based on the narrower abdomen, despite the genitalia being obscured by an opaque mass. Like many Baltic Amber taxa, *Eulonchiella* is closely related to a group of Afrotropical genera including *Dimacrocolus*, *Parahelle* and *Thyllis* (Hennig 1966), all with relatively complete wing venation. *Eulonchiella* can be differentiated from all other Philopotinae genera by the legs not being elongate, eyes not pilose, wing venation relatively complete, proboscis elongate and palpi being present.

### Quasi gen. n.

urn:lsid:zoobank.org:act:CD9618A4-E458-4B16-A7D6-0D188D77042E

http://species-id.net/wiki/Quasi

Figs 1A–C 3 5 

#### Type species.

*Quasi fisheri* sp. n.

#### Diagnosis.

Body shape arched; colouration non-metallic pale brown; head width slightly smaller than thorax width; shape hemispherical; postocular ridge and occiput rounded; posterior margin of eye rounded; eyes bare; three ocelli present, medial ocellus slightly smaller; position of antennae on head nearer to mouthparts; eyes contiguous above antennal base, not contiguous below; palpi absent; proboscis much shorter than head length; flagellum shape stylate, apex with terminal seta; thorax with postpronotal lobes enlarged, medially contiguous to form collar; subscutellum not enlarged, barely visible; legs with tibial spines absent; pulvilli present; legs not greatly elongated; wing hyaline, markings absent; costa ending in radial field; costal margin straight; humeral crossvein absent; radial veins meeting wing margin before wing apex; R1 slightly inflated distally at pterostigma; R2+3 present, reaching wing margin; R4+5 present as very short, single vein, not reaching wing margin; medial vein compliment with only one M vein present; discal cell absent; medial vein very short, not reaching wing margin; cell m3 absent; crossvein 2r-m absent; Cu reduced, not reaching wing margin; anal lobe not enlarged; alula well developed; abdomen smooth, shape rounded, cylindrical, similar width to thorax.

#### Etymology.

Derived from Latin *quasi*, appearing as if resembling; referring to the likeness of this species to members of *Terphis*.

#### Comments.

This genus is represented by only a single species *Quasi fisheri* sp. n. from Veracruz, Mexico. It is closely related to *Terphis* and *Philopota* based on reduction in wing veins. The position of the antennae, proximate to the reduced mouthparts, reduced wing venation and absence of abdominal tubercles readily differentiates this genus from all other philopotine genera.

### Quasi fisheri sp. n.

urn:lsid:zoobank.org:act:373C7B1F-51DF-4D7C-95FC-F91060433501

http://species-id.net/wiki/Quasi_fisheri

#### Type material.

**Holotype** male, MEXICO: Veracruz: Córdoba, 12-25.vii.1964, E. Fisher, D. Verity [18.896, -96.923] (CSCA).

#### Description.

Medium body size (male body: 6.0 mm), male wing almost as long as the body (male wing: 5.3 mm); *Head*. Eyes, antennae, face and occiput brown, occiput as narrow as the ocellar tubercle, ocelli brown, antennal tubercle brown and smaller than pedicel. *Thorax*. Postpronotal lobes, mesothorax, scutellum, subscutellum and coxae light brown with darker longitudinal markings, legs and lower calypter yellowish brown, pulvilli yellow, tarsal claws black, haltere yellow, wing hyaline with yellow veins. *Abdomen*. Tergites brown, with lateral margins yellow, sternites dark brown.

*Male genitalia*. The genitalia were not dissected because the holotype is the only specimen available. Genitalic dissection is not necessary to diagnose the genus, since it can be differentiated based on external characters.

#### Etymology.

This species is named in honor of Eric Fisher, the collector of the only known specimen of this unusual species.

### Schlingeriella gen. n.

urn:lsid:zoobank.org:act:99EAC1BE-4A6F-43E0-B61A-6460BF68694E

http://species-id.net/wiki/Schlingeriella

Figs 1D 6 7 

#### Type species.

*Schlingeriella irwini* sp. n.

#### Diagnosis.

Body shape arched; colouration non-metallic dark brown; head width much smaller than thorax (female) or slightly smaller than thorax (male); head spherical; postocular ridge and occiput extended posteriorly into slight ridge; posterior margin of eye rounded; eyes bare; position of antennae on head near middle of frons, slightly nearer to mouthparts; eyes contiguous above antennal base, not contiguous below; palpi present; proboscis longer than head; antennal flagellum stylate, apex with terminal seta; thorax with postpronotal lobes enlarged, medially contiguous to form collar; subscutellum enlarged; legs not greatly elongated; tibial spines absent; pulvilli present; wing hyaline, markings absent; costa ending in radial field; costal margin straight in both sexes; humeral crossvein absent; radial veins meeting wing margin before wing apex; R1 inflated distally at pterostigma; R2+3 present; R4+5 present as single vein, slightly curved anteriorly midway; veins M1, M2 and M3 present; discal cell absent; medial veins reaching wing margin (or nearly so); cell *m3* absent; crossvein 2r-m absent; Cu reduced, not reaching wing margin; anal lobe not enlarged; alula well developed; abdomen smooth, rounded, cylindrical in shape, similar width to thorax (male) or greatly rounded, inflated (female).

#### Etymology.

This genus is named in honor of Evert I. Schlinger, not only a collector of specimens of this species, but a foremost expert on world Acroceridae taxonomy and patron of dipterology.

#### Comments.

This genus is represented by only a single species (*Schlingeriella irwini* sp. n.) from New Caledonia. Winterton et al. (2007) included DNA sequences for this genus in their phylogenetic analysis of the family, placing it close to the New Zealand genus *Helle*. *Schlingeriella* gen n. can be differentiated from all other philopotine genera by a combination of the following characters: inflated vein R1 apically, medial veins mostly reaching the wing margin, absence of all wing cells except cell *br*, apilose eyes and elongate mouthparts. There is dramatic sexual dimorphism in body size, with females considerably larger than the diminutive males; males of this genus are some of the smallest acrocerids known.

### Schlingeriella irwini sp. n.

urn:lsid:zoobank.org:act:9AF204C7-FA7F-4DBC-B71D-4FFFCE367FB4

http://species-id.net/wiki/Schlingeriella_irwini

#### Genbank accessions.

AY144402.1, AY140881.1, AF539888.1

#### Type material.

**Holotype** male, New Caledonia: Riviere Bleue, refuse area, 700’, 28.xi.1992 E. & M. Schlinger, at *Scaveola* flower, prey of green crab spider [-22.112, 166.677] (MNHN) (EIS013911).

#### Paratypes.

New Caledonia: 3 males, 1 female, Riviere Bleue, same data as holotype (CAS, EIS013912, 013913) (CSCA, 013914, 013915); male, Riviere Bleue, 600’, 19.6 km on Riviere Bleue Road. M.T., 16-17.xi.1992, E. & M. Schlinger coll. (EIS013910); female, Riviere Bleue, 700’, Malaise, 6-16.xi.1992, E. & M. Schlinger, D. Webb; 1 male, 1 female [abdomen only], Mt. Nihgua, Nov. 2000, E. I. Schlinger & L. J. Boutin [voucher specimens from Winterton et al. (2007)] (EIS007431, male; EIS011170, female) (CAS); 1 female, Col d’Amieu Forestry Camp, 450m 17-18.x.1978, J. S. Dugdale, Malaise trap [-21.576, 165.740] (EIS013909) (CAS); 1 male, Mt. Ouin, 1100m, 9.xi.2002, C. Burwell & G. Monteith, pyrethrum, trees & logs (-22.016, 166.466) (QM).

#### Description.

Male with small body size (male body: 2.4 mm) and wing as long as the body (male wing: 2.5 mm), female with medium body size (female body: 4.4 mm) and wing longer than the body (female wing: 6.0 mm). *Head*. Eyes, occiput and ocellar tubercle dark brown, occiput wider than the face; ocelli shining light brown, antennal tubercle shining black, antennae light brown, face black, clypeus shining brown, as long as the antennae, proboscis yellow. *Thorax*. Uniform dark brown with short whitish pile; coxae yellow, legs dark yellow, femora with darker yellow-brown suffusion, lower calypter and haltere pale yellow, wings hyaline with brown veins. *Abdomen*. Dark brown; female tergites I-II entirely brown, tergites III-VI with the anterior half yellow and the posterior half brown, sternites brown.

#### Etymology.

This species is named in honour of Michael E. Irwin.

##### Key to world genera of living and fossil Philopotinae

**Table:** 

1	Wing venation reduced, with only one basal cell (br) present (Fig. 1B, D)	**7**
–	Wing venation relatively complete, with additional cells d, bm, cu-p and basal r4+5 present (Fig. 1E)	**2**
2	Palpi present	**4**
–	Palpi absent	**3**
3	Eyes densely pilose (South Africa and Madagascar)	*Thyllis* Erichson, 1840
–	Eyes very sparsely pilose or bare (Madagascar)	*Parahelle* Schlinger, 1961
4	Eyes pilose	**6**
–	Eyes apilose	**5**
5	Eyes contiguous below antennae; humeral crossvein present; vein R1 not inflated (Baltic amber) (Figs 1E-G, 2)	*Eulonchiella* Meunier, 1912
–	Eyes separate below antennae; humeral crossvein absent; vein R1 inflated at pterostigma (New Zealand)	*Helle* Osten Sacken, 1896
6	Legs relatively very long; male with tufted projection at the base of costa (Madagascar)	*Dimacrocolus* Schlinger, 1961
–	Legs of normal length; male without tufted projection at the base of costa (South America)	*Megalybus* Philippi, 1865
7	Eyes pilose	**12**
–	Eyes apilose	**8**
8	Mouthparts equal to, or longer than head length	**9**
–	Mouthparts much shorter than head length	**10**
9	Wing veins reaching wing margin; M2 not connected to M vein, unsclerotized and discontinuous basally; vein R1 inflated at pterostigma (New Caledonia) (Figs 1D, 6–7)	*Schlingeriella* gen. n.
–	Wing veins not reaching wing margin (Hennig 1966: Fig. 11), M2 originating on M vein, sclerotized and continuous basally; vein R1 not inflated at pterostigma (Baltic amber)	*Prophilopota* Hennig, 1966
10	Three pairs of tubercles present on segments II - IV of abdomen; occiput extended posteriorly to form an acute ridge (South America)	*Terphis* Erichson, 1840
–	Abdomen without tubercles; occiput rounded, not extended posteriorly	**11**
11	Antenna on ventral side of head, adjacent to mouthparts; abdomen conical (Mexico) (Figs 1A-C, 3–5)	*Quasi* gen. n.
–	Antennae on lower front side of head, but not adjacent to mouthparts; abdomen rounded (Africa)	*Africaterphis* Schlinger, 1968
12	Large hemispherical head; posterior margin of eye emarginate; mouthparts shorter than head; occiput rounded; postpronotal lobes proximate but not contiguous medially (Baltic amber)	*Archaeterphis* Hauser & Winterton, 2007
–	Head smaller and almost spherical, eye not emarginate posteriorly; mouthparts elongate, longer than head, occiput extended posteriorly to form acute ridge; postpronotal lobes contiguous medially	**13**
13	Palpi present (Palaearctic)	*Oligoneura* Bigot, 1878
–	Palpi absent (Neotropical)	*Philopota* Wiedemann, 1830

## Systematics of Philopotinae

While two groups can be differentiated within Philopotinae based on reduction of wing venation, three clades have been identified by Winterton et al. (2007) largely corresponding to three biogeographical regions.

*Philopota* genus group– This genus group comprises *Africaterphis* from Africa, the Palaearctic *Oligoneura*, *Archaeterphis* and *Prophilopota*, and new world genera *Philopota*, *Megalybus*, *Quasi* gen n. and *Terphis*. *Archaeterphis* is a very distinctive genus, closely related to the extant genus *Africaterphis* (Hauser & Winterton, 2007). *Prophilopota* is presumably more closely related to *Oligoneura*, since both genera share the presence of maxillary palpi and similar shape of the antennal tubercle (Schlinger, 1971). *Quasi* gen n. is closely related to *Philopota* and in particular, *Terphis*. This genus shares with *Terphis* the insertion of the antennae on the lower side of head, reduced mouthparts, presence of relatively well-developed subscutellum and substantial reduction of the wing venation, the latter being less reduced in *Philopota*. In addition, both *Philopota *and *Quasi* gen n.lack the abdominal tubercles present in *Terphis*, and share a conical abdomen, instead of a swollen one that characterizes *Terphis*. Genera in the *Philopota* genus group are found in the northern and southern hemispheres with greater diversity in the New World (four genera). All genera in this group have reduced wing venation except *Megalybus*, the sister genus to the clade (Winterton et al. 2007).

*Helle* genus group– *Schlingeriella* gen n. was included in the study by Winterton et al. (2007) as “undescribed genus New Caledonia”. It is closely related to the New Zealand genus *Helle*, since they both have apilose eyes, well developed palpi, elongate mouthparts and an inflation of the vein R1 at the pterostigma. *Schlingeriella* gen n. is differentiated from *Helle* by its small body size and the reduced wing venation.

*Thyllis* genus group– This group contains genera with complete wing venation including three Afrotropical genera (*Dimacrocolus*, *Parahelle* and *Thyllis*) and the Palaearctic genus *Eulonchiella*. *Dimacrocolus* and *Parahelle* are endemic to Madagascar while *Thyllis* is found in both Madagascar and South Africa.

## Supplementary Material

XML Treatment for Eulonchiella

XML Treatment for Quasi

XML Treatment for Quasi fisheri

XML Treatment for Schlingeriella

XML Treatment for Schlingeriella irwini
